# White and gray matter alterations in bipolar I and bipolar II disorder subtypes compared with healthy controls – exploring associations with disease course and polygenic risk

**DOI:** 10.1038/s41386-024-01812-7

**Published:** 2024-02-08

**Authors:** Katharina Thiel, Hannah Lemke, Alexandra Winter, Kira Flinkenflügel, Lena Waltemate, Linda Bonnekoh, Dominik Grotegerd, Katharina Dohm, Tim Hahn, Katharina Förster, Philipp Kanske, Jonathan Repple, Nils Opel, Ronny Redlich, Friederike David, Andreas J. Forstner, Frederike Stein, Katharina Brosch, Florian Thomas-Odenthal, Paula Usemann, Lea Teutenberg, Benjamin Straube, Nina Alexander, Hamidreza Jamalabadi, Andreas Jansen, Stephanie H. Witt, Till F. M. Andlauer, Andrea Pfennig, Michael Bauer, Igor Nenadić, Tilo Kircher, Susanne Meinert, Udo Dannlowski

**Affiliations:** 1 Institute for Translational Psychiatry, University of Münster, Münster, Germany; 2 Translational Psychotherapy, Institute of Psychology, University of Göttingen, Göttingen, Germany; 3 Clinical Psychology and Behavioral Neuroscience, Faculty of Psychology, Technische Universität Dresden, Dresden, Germany; 4 Department for Psychiatry, Psychosomatic Medicine and Psychotherapy, University Hospital Frankfurt, Goethe University, Frankfurt, Germany; 5 Department of Psychiatry, Jena University Hospital/Friedrich-Schiller-University Jena, Jena, Germany; 6 German Center for Mental Health (DZPG), Halle-Jena-Magdeburg, Germany; 7 Department of Psychology, University of Halle, Halle, Germany; 8 Center for Intervention and Research on adaptive and maladaptive brain circuits underlying mental health (C-I-R-C), Jena-Magdeburg-Halle, Halle, Germany; 9 Institute of Human Genetics, University of Bonn, School of Medicine & University Hospital Bonn, Bonn, Germany; 10 Institute of Neuroscience and Medicine (INM-1), Research Center Jülich, Jülich, Germany; 11 Department of Psychiatry and Psychotherapy, University of Marburg, Marburg, Germany; 12 Center for Mind, Brain and Behavior (CMBB), University of Marburg, Marburg, Germany; 13 Core-Facility Brainimaging, Faculty of Medicine, University of Marburg, Marburg, Germany; 14 Central Institute of Mental Health, Medical Faculty Mannheim, Heidelberg University, Mannheim, Germany; 15 Department of Neurology, Klinikum rechts der Isar, School of Medicine, Technical University of Munich, Munich, Germany; 16 Department of Psychiatry and Psychotherapy, Faculty of Medicine, TU Dresden University of Technology, Dresden, Germany; 17 Institute of Translational Neuroscience, University of Münster, Münster, Germany

**Keywords:** Diagnostic markers, Bipolar disorder, Translational research, Genetics research

## Abstract

Patients with bipolar disorder (BD) show alterations in both gray matter volume (GMV) and white matter (WM) integrity compared with healthy controls (HC). However, it remains unclear whether the phenotypically distinct BD subtypes (BD-I and BD-II) also exhibit brain structural differences. This study investigated GMV and WM differences between HC, BD-I, and BD-II, along with clinical and genetic associations. *N* = 73 BD-I, *n* = 63 BD-II patients and *n* = 136 matched HC were included. Using voxel-based morphometry and tract-based spatial statistics, main effects of group in GMV and fractional anisotropy (FA) were analyzed. Associations between clinical and genetic features and GMV or FA were calculated using regression models. For FA but not GMV, we found significant differences between groups. BD-I patients showed lower FA compared with BD-II patients (*p*_tfce-FWE_ = 0.006), primarily in the anterior corpus callosum. Compared with HC, BD-I patients exhibited lower FA in widespread clusters (*p*_tfce-FWE_ < 0.001), including almost all major projection, association, and commissural fiber tracts. BD-II patients also demonstrated lower FA compared with HC, although less pronounced (*p*_tfce-FWE_ = 0.049). The results remained unchanged after controlling for clinical and genetic features, for which no independent associations with FA or GMV emerged. Our findings suggest that, at a neurobiological level, BD subtypes may reflect distinct degrees of disease expression, with increasing WM microstructure disruption from BD-II to BD-I. This differential magnitude of microstructural alterations was not clearly linked to clinical and genetic variables. These findings should be considered when discussing the classification of BD subtypes within the spectrum of affective disorders.

## Introduction

Bipolar disorder (BD) is a heterogeneous mood disorder that is divided into two phenotypically distinct subtypes based on DSM (Diagnostic and Statistical Manual of Mental Disorders) diagnostic criteria [1]. While subtype I (BD-I) is characterized by periods of mania, which may alternate with depressive episodes, subtype II (BD-II) is diagnosed when hypomanic and depressive episodes are present but no episodes of full mania. Beyond diagnostic criteria, differences in the long-term disease course between subtypes have been found, with more (hypo-)manic episodes [2, 3] and hospitalizations [4, 5] in BD-I and more frequent and prolonged depressive episodes [3, 4, 6] in BD-II. Depending on the subtype, different treatment approaches may be more beneficial [4, 7–10]. Further, differential diagnosis of subtypes is commonly based on phenotypic characteristics and clinical assessment of the degree of impairment, with a clear differentiation only being possible if certain characteristics are present [11]. Therefore, a key challenge for clinicians remains the distinction of BD subtypes [12, 13]. This underlines the importance of investigating other, potentially meaningful markers to enhance subtype diagnosis and treatment response. Studying neuronal [14] and genetic [15, 16] correlates contributes to a more detailed subtype characterization and could provide additional clinical and therapeutic benefits [12, 17].

Previous neuroimaging research showed widespread white and gray matter abnormalities in BD patients compared with healthy controls (HC) [18–23]. However, some studies included one subtype only [18, 23–26] or did not differentiate between subtypes in their analyses [19, 27–31]. Those few studies that directly compared subtypes yielded mainly inconclusive results [20, 21, 32–40]: Diffusion tensor imaging (DTI) studies, which usually focus on fractional anisotropy (FA) as a quantitative measure of WM microstructure or integrity, have yielded conflicting results when conducting direct comparisons between subtypes, with some finding reduced FA in the temporal and frontal pathways in BD-I [34, 35, 38] and others in BD-II [32, 39]. Although these studies only allow tentative conclusions due to consistently small samples (mostly *n* < 30 per group) and varying methods, current evidence rather points to more severe WM microstructural impairments in BD-I compared with BD-II [41]. Regarding gray matter volumes (GMV), some studies found lower volumes in temporal [37, 40, 42], (pre-)frontal [37, 40, 42] and posterior cingulate regions [37] and in the putamen [33] in BD-I compared with BD-II, while others found no GMV differences between subtypes [20, 21, 36, 43, 44]. In conclusion, although there seems to be preliminary data attesting more pronounced WM and GM changes in BD-I compared with BD-II e.g. [35, 37, 38, 42], the evidence is inconsistent and it remains unclear whether existing neuroimaging findings on BD can be generalized to both subtypes.

Going one step further, the issue is whether neurobiological alterations support the categorical classification into clinical subtypes according to DSM, which has so far been based on phenotypic markers only [45]. Even though these phenotypic features are dimensional in nature, they are categorized based on the severity of their expression resulting in the two discrete BD subtypes. Therefore, neurobiological alterations may form a continuum, representing the subtypes as one clinical entity with varying severity. This would be captured by a dimensional diagnostic approach, where neurobiological alterations are associated with dimensional clinical features rather than discrete diagnostic categories [46, 47].

Underlying genetics provide evidence that fit both approaches. Based on the latest genome-wide association study (GWAS), BD subtypes share a large amount of genetic composition, showing a strong between-subtypes correlation of *r* = 0.85, while correlations with other mental disorders such as schizophrenia (SZ) or MDD were weaker (all *r* ≤ 0.66) [15]. In contrast, the strength of the GWAS correlations with other mental disorder differed between subtypes, with a greater correlation between BD-II and MDD, and between BD-I and SZ [15, 48, 49]. Moreover, heritability estimates based on twin studies [48, 50] and GWAS-based h^2^_SNP_ heritability was estimated to be higher for BD-I compared to BD-II [15, 51]. Beyond differences in measures of heritability, identification of distinct loci might be related to subtype-specific symptomatology [15]. BD-I thus might have a stronger genetic component than BD-II.

A higher genetic load, which was also related to a more severe course of BD across subtypes [52], could also be linked to brain structural alterations [53–55]. Despite evidence for subtype-specific neuroanatomy and genetics, the relationship between these features in BD subtypes remains uninvestigated. Exploring the effects of genetic factors on brain structure altered in BD subtypes may provide insight into the neural mechanisms by which genetic variation has an impact on the disease at the psychopathological level.

To date, we are not aware of any study investigating subtype-specific differences in GMV and WM within the same sample subdivided by BD type. This study focuses on WM and GMV differences among BD-I, BD-II, and HC, aiming to investigate the neurobiological underpinnings of conventional BD subtype categories. Given previous heterogeneous neuroimaging findings [20, 21, 32–40], we employed a whole-brain approach. Rather than adhering to diagnostic categories, we also took a dimensional approach, exploring other potential subtype-specifying factors in relation to neurobiology. Adding this perspective, we aim to contribute to a more nuanced understanding of the neurobiological basis of the subtypes of BD.

First, we expected a decrease in WM integrity and GMV in BD patients, resulting in the following pattern of group differences: BD-I < BD-II < HC (categorical perspective). Second, we exploratively examined potential relationships between white and gray matter, BD polygenic risk scores (PRS) and various clinical characteristics (dimensional perspective).

## Materials and methods

### Participants

This study included *n* = 136 patients with BD and *n* = 136 HC from the Marburg-Münster-Affective-Cohort-Study (MACS; see ref. [56] for a general description). The data in the current study are a subsample of a previously published analysis by our group on WM microstructural differences between healthy, depressed, and bipolar individuals regardless of subtype [22]. Recruitment was conducted via newspaper advertisement and flyers, and in psychiatric hospitals. Inclusion criteria for HC were the absence of any current or lifetime psychiatric disorder, whereas BD patients required a current or lifetime diagnosis of bipolar disorder. The presence or absence of mental disorders was assessed by trained personnel with the Structured Clinical Interview (SCID-I) [57] according to the DSM-IV-TR criteria [58]. Based on this, BD patients were grouped into Bipolar I (BD-I, *n* = 73; *n* = 42 female, *M*_*age*_ = 41.77, *SD*_age_ = 11.51) and Bipolar II (BD-II, *n* = 63; *n* = 33 female, *M*_age_ = 40.48, *SD*_*age*_ = 12.56) subtypes. General exclusion criteria comprised usual magnetic resonance imaging (MRI) contraindications, head trauma, and any history of neurological, cardiovascular, or other severe medical conditions (e.g., cancer, infections, and autoimmune disease). Further exclusion criteria for BD patients were a lifetime diagnosis of alcohol or substance dependence (other than tetrahydrocannabinol dependence), while any current intake of psychotropic medication resulted in exclusion from the study for HC. All participants were aged between 18-65 years and HC and BD patients were matched according to age, sex, and study sites using the MatchIt package in R [59].

During the clinical interview, information about current symptomatology, lifetime course of disease, and psychopharmacological treatment was collected. The 21-item Hamilton Depression Rating Scale [60] and the Young Mania Rating Scale [61] were used to measure current depressive and (hypo-)manic symptoms, respectively. Disease course variables including number and cumulative duration of depressive and (hypo-)manic episodes and psychiatric hospitalizations, time since first symptoms (measured by age minus age of onset), and time since first psychiatric hospitalization were assessed by patients’ self-reports. Current psychopharmacological medication intake was assessed through a previously used composite score, the medication load index [62] (Supplement 1). PRS for bipolar disorder were calculated using genome-wide genotype data and the summary statistics of a recent GWAS of BD (more information in Supplement 2) [15]. Sociodemographic, clinical, and genetic information are provided in Table 1.

**Table 1:** Demographic and clinical characteristics of BD-I and BD-II patients and HC.

	BD-I (*n* = 73)	BD-II (*n* = 63)	Tests for both patient groups		HC (*n* = 136)	Tests for all three groups	
	Mean ± SD	Mean ± SD	Test-statistic	*p*-value	mean ± SD	Test-statistic	*p*-value
**Descriptive characteristics**							
Age	41.77 ± 11.51	40.48 ± 12.56	0.625^a^	0.533	42.46 ± 12.92	0.440^d^	0.645
Sex (f/m)	42/31	33/30	0.363^b^	0.547	77/59	0.424^b^	0.809
Education (years)	13.83 ± 2.76	14.22 ± 2.81	0.662^c^	0.508	14.26 ± 2.76	1.154^e^	0.562
**Questionnaires**							
HDRS scores^f^	6.82 ± 6.52 (*n* = 72)	8.30 ± 6.48	1.305^c^	0.192	1.02 ± 1.48	105.351^e^	<0.001
YMRS scores	3.61 ± 5.58	3.87 ± 5.51	0.176^c^	0.860	0.49 ± 1.14	51.083^e^	<0.001
CTQ scores^i^	43.61 ± 15.65	41.95 ± 13.14	0.655^a^	0.513	33.02 ± 10.01	21.023^d^	<0.001
**Psychiatric medication**							
Medication load index^i^	2.64 ± 1.90	2.49 ± 2.20	−0.832^c^	0.405	n/a	n/a	n/a
None (yes/no)	9/64	13/50	1.721^b^	0.190	n/a	n/a	n/a
Antidepressants (yes/no)	25/48	31/32	3.124^b^	0.077	n/a	n/a	n/a
Antipsychotics (yes/no)	43/30	21/42	8.875^b^	0.003	n/a	n/a	n/a
Anticonvulsives (yes/no)	23/50	21/42	0.050^b^	0.820	n/a	n/a	n/a
Lithium (yes/no)	25/48	13/50	3.112^b^	0.078	n/a	n/a	n/a
**Clinical characteristics**							
Remission status (acute/partly-/fully remitted)^f^	30/21/21 (*n* = 72)	26/12/18 (*n* = 56)	1.353^b^	0.508	n/a	n/a	n/a
Polarity of the current or last episode (depressive/manic/mixed)^f^	44/22/5 (*n* = 71)	37/15/0 (*n* = 52)	4.092^b^	0.129	n/a	n/a	n/a
Lifetime psychotic symptoms (yes/no)^f,h,i^	44/27 (*n* = 71)	17/45 (*n* = 62)	15.915^b^	<0.001	n/a	n/a	n/a
Number of depressive episodes^f,i^	7.96 ± 7.79 (*n* = 68)	7.60 ± 7.66 (*n* = 60)	−0.607^c^	0.544	n/a	n/a	n/a
Duration of depressive episodes (months)^f^	53.10 ± 86.84 (*n* = 53)	48.84 ± 53.71 (*n* = 58)	1.122^c^	0.262	n/a	n/a	n/a
Number of (hypo-)manic episodes^f,i^	5.46 ± 5.90 (*n* = 68)	6.59 ± 10.27 (*n* = 59)	−0.492^c^	0.623	n/a	n/a	n/a
Duration of (hypo-)manic episodes (months)^f^	15.66 ± 30.75 (*n* = 58)	19.46 ± 36.68 (*n* = 53)	−0.251^c^	0.801	n/a	n/a	n/a
Time since first symptoms (months)^f,i^	213.00 ± 134.24 (*n* = 72)	196.00 ± 150.74	−0.980^c^	0.327	n/a	n/a	n/a
Time since first psychiatric hospitalization (months)^f,i^	179.79 ± 131.78 (*n* = 70)	126.34 ± 120.08 (*n* = 62)	−2.643^c^	0.008	n/a	n/a	n/a
Number of inpatient treatments^f,i^	4.36 ± 3.43 (*n* = 70)	3.03 ± 2.69 (*n* = 62)	−2.596^c^	0.009	n/a	n/a	n/a
Duration of inpatient treatments (weeks)^f^	39.22 ± 33.44 (*n* = 68)	25.96 ± 32.48 (*n* = 61)	−3.044^c^	0.002	n/a	n/a	n/a
Lifetime psychiatric comorbidity (yes/no)^f^	25/48	29/34	1.962^b^	0.161	n/a	n/a	n/a
**Genetics**							
PRS-CS-auto (mean *φ* = 1.29 × 10^−4^) for BD^g^	0.50 ± 0.95 (*n* = 67)	0.10 ± 1.03 (*n* = 60)	2.26^a^	0.026	−0.32 ± .89 (*n* = 125)	17.046^d^	<0.001
**Body mass index**^i^	28.8 ± 5.39	25.99 ± 5.08	3.039^a^	0.003	25.07 ± 4.32	13.034^d^	<0.001

*BD-I* bipolar disorder subtype I, *BD-II* bipolar disorder subtype 2, *CTQ* childhood trauma questionnaire (Wingenfeld et al. [106]), *HC* healthy controls, *HDRS* Hamilton Depression Rating Scale, *YMRS* Young Mania Rating Scale, *SD* standard deviation, *n/a* not applicable.

^a^Two-Sample *t*-test.

^b^Pearson *χ*²-test.

^c^Mann–Whitney-*U*-test.

^d^One-way analysis of variance (ANOVA) *F*-test.

^e^Kruskal–Wallis-test.

^f^Not all participants provided the necessary information.

^g^Polgygenic risk score for bipolar disorder according to Mullins et al. [15] (z-scaled).

^h^Obtained during clinical interviews based on patients self-reports.

^i^Included either as covariate in the analyses of group differences (analyses 2.a) between BD subtypes or as variable of interest in the linear regression analyses (analyses 2.b).

The study was approved by the Ethics Committees of the Medical Faculties, University of Marburg (AZ: 07/14) and University of Münster (2014-422-b-S), in accordance with the Declaration of Helsinki. All participants received financial compensation and provided written informed consent prior to participation.

### Image acquisition and preprocessing

3 T whole body MRI scanners (Marburg: Tim Trio, Siemens, Erlangen, Germany; Münster: Prisma fit, Siemens, Erlangen, Germany) were used for acquisition of MRI data. All images underwent quality checks according to the quality check protocol of the MACS study [63]. Due to a body-coil change at the Marburg site, we controlled for three different scanner settings (Münster, Marburg body-coil pre, Marburg body-coil post) in all analyses using two dummy-coded variables with Münster as reference category as previously recommended [63].

#### White matter microstructure (DTI)

DTI data acquisition, preprocessing and quality assurance followed published protocols and were extensively described elsewhere [22, 63]. Preprocessing and analyses were implemented in FSL6.0.1 (http://fsl.fmrib.ox.ac.uk/fsl/fslwiki/) [64–66]. For details on DTI acquisition parameters, quality assurance of the data and preprocessing steps see Supplement 3. As the last step, a diffusion tensor model was fitted at each voxel using “DTIFIT” within FMRIB’s Diffusion Toolbox (FDT) [67] and FA, mean diffusivity (MD), radial diffusivity (RD) and axial diffusivity (AD) were calculated for each voxel per participant. FA is a measure of the directionality of water diffusion on a scale from 0 (indicating isotropic diffusion) to 1 (indicating completely anisotropic diffusion) [68]. See Supplement 3 for more information on MD, RD, and AD measures.

#### Gray matter volumes (GMV)

High-resolution T1-weighted structural images were collected using three-dimensional fast gradient echo sequences (MPRAGE). Details on acquisition of T1 data for GMV analyses are provided in Supplement 4 and have been described elsewhere [69]. Preprocessing of T1-weighted images was conducted using a default pipeline implemented in the CAT12-toolbox (v1720). Steps included bias-correction, tissue classification, realignment and spatial normalization to MNI space using the Geodesic Shooting algorithm. Data were smoothed with an 8 mm full width half maximum Gaussian kernel.

### Statistical analyses

Descriptive and clinical variables between BD patients and HC were analyzed in IBM SPSS Statistics 27 (SPSS Inc., Chicago, IL, USA; Table 1).

#### White matter microstructure (DTI)

Analysis of DTI data was performed using TBSS [70], a technique designed to reduce registration misalignment (Supplement 3). Voxel-wise statistical analyses were performed by using the nonparametric permutation testing implemented in “randomize” from FSL [71] with 5000 permutations. Threshold-Free Cluster Enhancement (TFCE) was applied to obtain cluster-wise statistics corrected for multiple comparisons [72]. Significance was determined using the 95th percentile of the null distribution of permutated input data of the maximum TFCE scores, allowing to correct estimated cluster sizes for family-wise error (FWE) at *p* < 0.05. The significant effect mask was placed over the raw diffusion metrics maps of each subject and the values of the respective voxels were extracted and averaged, using “fslstats” from FSL. The mean diffusion metric value for each subject from all voxels of the significant cluster was used for visualization in scatterplots.

The results for WM focus on FA. However, to support the interpretation of these results, the same registration steps and analyses were also performed for the other DTI metrics MD, RD, and AD (Supplement 5).

#### Gray matter volumes (GMV)

Statistical analyses of GMV were performed on the whole-brain level using Statistical Parametric Mapping (SPM12, Wellcome Department of Cognitive Neurology, London, UK, v7771) with an absolute threshold masking of 0.1. TFCE, as implemented in the TFCE-toolbox, with 5000 permutations per test was applied (http://dbm.neuro.uni-jena.de/tfce, Version r210). The significance threshold was set to *p* < 0.05 FWE corrected.

All DTI and GMV analyses included the covariates age, sex, total intracranial volume (TIV), site, and scanner settings (body-coil pre, body-coil post with Münster as reference category). The following analyses were conducted:

1. To examine brain structural and microstructural differences between the diagnostic groups (HC, BD-I, BD-II), we first performed *F*-tests. Subsequently, post-hoc pairwise *t*-contrasts were calculated. Effect sizes were calculated based on the mean *t*-value of all significant voxels provided by FSL or SPM and respective sample sizes [73].
2. To investigate putative effects of clinical characteristics, we performed additional analyses:
  - In case of significant effects in the contrast BD-II > BD-I in the main analyses (step 1), we repeated the group comparisons by separately including clinical variables (e.g. number of depressive and (hypo-)manic episodes, psychiatric hospitalization, psychopharmacological medication and PRS for BD) potentially related to differences between BD subtypes as additional nuisance variables. An overview of all included clinical variables can be found in Table 1.
  - To further determine which clinical characteristics influence brain structural alterations, associations between the clinical variables mentioned in a) and FA or GMV were calculated for the whole BD sample and per subtype (see Tables S5 and S6) using linear regression models. These analyses were calculated irrespective of significant group differences in step 1. Bonferroni correction for the ten regression analyses was applied, resulting in a significance threshold of *p* < 0.005.

## Results

### Brain structural differences between HC, BD-I and BD-II

#### White matter microstructure (DTI)

**Fig. 1:**
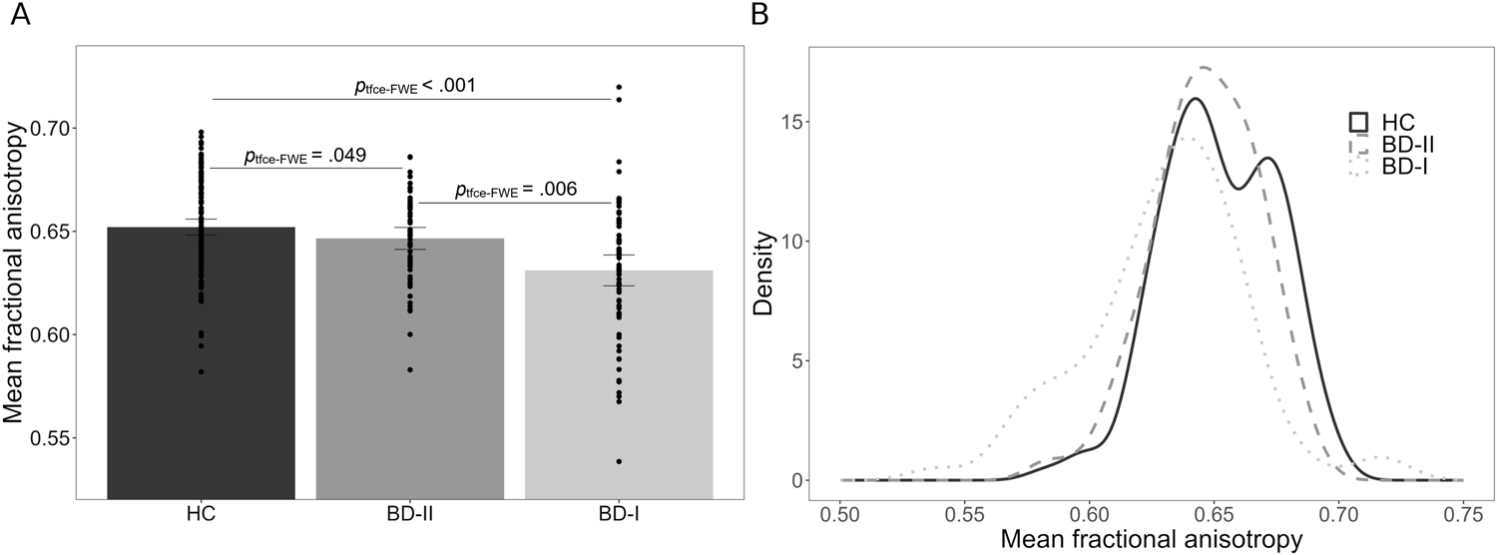
Main effect of diagnosis on FA. **A** Mean fractional anisotropy (FA) across healthy controls (HC), bipolar disorder type I (BD-I) and bipolar disorder type II (BD-II). The mean FA value was obtained from FA values of all the voxels that showed a significant main effect of diagnosis (*p*_tfce-FWE_ < 0.05). Error bars represent 95% confidence intervals. *p* values were obtained from pairwise post-hoc *t*-contrasts. **B** Density estimation plots of FA values showing distributional overlap between HC, BD-I and BD-II.

**Fig. 2:**
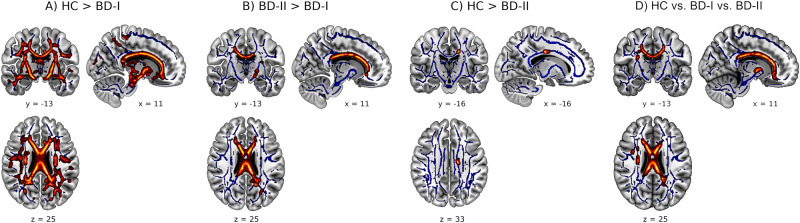
Differences in FA between HC, BD-I and BD-II. Differences in fractional anisotropy (FA) between healthy controls (HC), bipolar disorder type I (BD-I), and bipolar disorder type II (BD-II). **A** Higher FA in HC compared with BD-I. **B** Higher FA in BD-II compared with BD-I. **C** Higher FA in HC compared with BD-II. **D** Differences in FA between the three groups HC, BD-I and BD-II (*F*-Test). Coordinates are given in MNI space. Highlighted areas represent voxels (using FSL’s ‘fill’ command for better visualization), where significant differences between groups (*p*_tfce-FWE_ < 0.05) were detected.

The *F*-contrast revealed a significant main effect of diagnosis in FA (*p*_tfce-FWE_ < 0.001, total *k* = 7028 voxels in seven clusters, Fig. 1, Table S1). Pairwise post-hoc *t*-contrasts revealed significantly lower FA values in BD-I patients compared with HC in one large bilateral cluster (*d* = 0.25, *p*_tfce-FWE_ < 0.001, *k* = 45712 voxels, Fig. 2A) as well as compared with BD-II patients (*d* = 0.36, *p*_tfce-FWE_ = 0.006, *k* = 6418 voxels in seven clusters, Fig. 2B). Both effects were most probably located in the forceps minor of the corpus callosum (CC), with almost all other major fiber tracts also affected when BD-I and HC were compared (Table S2). BD-II patients also showed significant lower FA values compared with HC in two small clusters in the left body of the CC (*d* = 0.56, *p*_tfce-FWE_ = 0.049, *k* = 27 voxels in two clusters, Fig. 2C). There was also a significant main effect of diagnosis for RD and MD, reflected in RD by significantly higher scores for BD-I compared with HC and BD-II, and in MD by significantly higher scores only for BD-I compared with HC. No effects were found for AD (Supplement 5).

#### Gray matter volumes (GMV)

In the whole-brain analysis no significant main effect of diagnosis was found (*p*_tfce-FWE_ = 0.509). Exploratory pairwise comparisons between diagnoses on the whole-brain level, uncorrected at *p* < 0.001 with a cluster threshold of *k* = 50 voxels pointed towards putative GMV alterations in parietal, frontal and parahippocampal/fusiform regions in the expected pattern of BD-I < BD-II < HC (Table S3).

### Additional analyses

#### Brain structural differences between BD-I and BD-II subtypes correcting for clinical variables and polygenic risk

##### White matter microstructure (DTI)

Analyses revealed a stably lower FA in BD-I vs. BD-II even when additionally correcting for number of depressive episodes (*d* = 0.36, *p*_tfce-FWE_ = 0.004, *k* = 6832 voxels), number of (hypo-)manic episodes (*d* = 0.36, *p*_tfce-FWE_ = 0.005, *k* = 8061 voxels), number of psychiatric hospitalizations (*d* = 0.31, *p*_tfce-FWE_ = 0.003, *k* = 16683 voxels), time since first symptoms (*d* = 0.36, *p*_tfce-FWE_ = 0.007, *k* = 6689 voxels), time since first psychiatric hospitalization (*d* = 0.32, *p*_tfce-FWE_ = 0.007, *k* = 17,880 voxels), and lifetime psychotic symptoms (*d* = 0.37, *p*_tfce-FWE_ = 0.013, *k* = 5202 voxels). Similarly, the observed pattern of results did not change when correcting the models for PRS for BD (*d* = 0.38, *p*_tfce-FWE_ = 0.020, *k* = 4742 voxels), childhood adversity (*d* = 0.32, *p*_tfce-FWE_ = 0.005, *k* = 17,248 voxels), body mass index (*d* = 0.53, *p*_tfce-FWE_ = 0.038, *k* = 307 voxels) or medication load (*d* = 0.36, *p*_tfce-FWE_ = 0.005, *k* = 6745 voxels, additional analyses for different types of medication are provided in Table S4). Affected tracts consistently included the forceps minor and major of the CC as well as the anterior thalamic radiation (Table S2). The significant increase in RD for BD-I compared with BD-II also proved stable in these additional analyses, except when PRS for BD, body mass index and psychotic symptoms were included as covariates (Supplement 5).

#### Associations with clinical variables and polygenic risk

##### White matter microstructure (DTI)

There was a significant positive associations between the time since first psychiatric hospitalization and FA (*p*_tfce-FWE_ = 0.021), which, however, did not survive Bonferroni correction (Table 2). RD, MD and AD showed a negative association with this variable, which only survived Bonferroni correction in the case of MD (Supplement 5).

**Table 2:** Associations between clinical variables and FA within all BD patients.

Variable	Negative	Positive
Number of depressive episodes	*p*_tfce-FWE_ = 0.057	*p*_tfce-FWE_ = 0.737
Number of (hypo-) manic episodes	*p*_tfce-FWE_ = 0.05	*p*_tfce-FWE_ = 0.93
Number of psychiatric hospitalizations	*p*_tfce-FWE_ = 0.938	*p*_tfce-FWE_ = 0.108
Time since first psychiatric hospitalization	*p*_tfce-FWE_ = 0.989	*p*_tfce-FWE_ = 0.021
Time since first symptoms	*p*_tfce-FWE_ = 0.532	*p*_tfce-FWE_ = 0.505
Psychotic symptoms (yes vs. no)^a^	*p*_tfce-FWE_ = 0.472	
Medication load	*p*_tfce-FWE_ = 0.616	*p*_tfce-FWE_ = 0.318
PRS-CS-auto (mean *φ* = 1.29 × 10^−4^) for BD	*p*_tfce-FWE_ = 0.971	*p*_tfce-FWE_ = 0.278
Childhood adversity^b^	*p*_tfce-FWE_ = 0.081	*p*_tfce-FWE_ = 0.889
Body Mass Index	*p*_tfce-FWE_ = 0.056	*p*_tfce-FWE_ = 0.718

*p* values are considered significant at *p* < 0.005, correcting for multiple testing by the Bonferroni procedure, ^a^according to pairwise comparison (t-test) comparing patients with and without lifetime psychotic symptoms across both BD subtype groups, ^b^measured by the Childhood Trauma Questionnaire (CTQ).

*BD* bipolar disorder, *BD-I* bipolar disorder subtype I, *BD-II* bipolar disorder subtype 2, *FA* fractional anisotropy, *PRS* polygenic risk score.

##### Gray matter volumes (GMV)

Regression analyses between clinical variables and GMV revealed no significant associations (all *p*_tfce-FWE_ > 0.072, Table 3). The results of the additional regression analyses between clinical variables and brain structure in both subtype groups separately can be found in Table S5 (DTI metrics) and Table S6 (GMV).

**Table 3:** Associations between clinical variables and GMV within all BD patients.

Variable	Negative	Positive
Number of depressive episodes	*p*_tfce-FWE_ = 0.162	*p*_tfce-FWE_ = 0.589
Number of (hypo-) manic episodes	*p*_tfce-FWE_ = 0.427	*p*_tfce-FWE_ = 0.831
Number of psychiatric hospitalizations	*p*_tfce-FWE_ = 0.132	*p*_tfce-FWE_ = 0.999
Time since first psychiatric hospitalization	*p*_tfce-FWE_ = 0.416	*p*_tfce-FWE_ = 0.785
Time since first symptoms	*p*_tfce-FWE_ = 0.329	*p*_tfce-FWE_ = 0.646
Age of Onset	*p*_tfce-FWE_ = 0.646	*p*_tfce-FWE_ = 0.329
Psychotic symptoms (yes vs. no)^a^	*p*_tfce-FWE_ = 0.356	
Medication Load	*p*_tfce-FWE_ = 0.072	*p*_tfce-FWE_ = 0.999
PRS-CS-auto (mean φ = 1.29×10-4) for BD	*p*_tfce-FWE_ = 0.571	*p*_tfce-FWE_ = 0.314
Childhood Adversity^b^	*p*_tfce-FWE_ = 0.098	*p*_tfce-FWE_ = 0.988
Body Mass Index	*p*_tfce-FWE_ = 0.546	*p*_tfce-FWE_ = 0.621

*p*-values are considered significant at *p* < 0.005, correcting for multiple testing by the Bonferroni procedure, ^a^according to pairwise comparison (t-test) comparing patients with and without lifetime psychotic symptoms across both BD subtype groups, ^b^measured by the Childhood Trauma Questionnaire (CTQ).

*BD* bipolar disorder, *BD-I* bipolar disorder subtype I, *BD-II* bipolar disorder subtype 2, *GMV* gray matter volumes, *PRS* polygenic risk score.

## Discussion

This study examined brain structural differences between patients with BD types I and II and HC, including white and gray matter, PRS for BD and disease course data, attempting to provide a more profound comparison of the subtypes. As the main result, our analyses revealed group differences with the expected pattern of BD-I < BD-II < HC regarding FA as a measure of WM integrity, whereas no group differences were found for GMV. Secondly, the group differences in WM microstructure were not significantly affected by prior disease course or PRS for BD and thus seem to be nearly independent of other clinical factors or underlying genetics. Finally, we found no associations between brain structure and these clinical or genetic parameters in either BD-I or BD-II.

Bringing some clarity to the heterogeneity of previous neuroimaging findings [20, 21, 32, 34, 35, 42], we demonstrated significantly lower FA, along with higher RD, in BD-I patients compared with BD-II. This finding was accompanied by a markedly different extent of impairment when compared with HC: Whereas BD-I patients showed widespread alterations in WM affecting all major WM tracts (including the CC), BD-II patients differed from HC only in a small local cluster in the CC. The FA reduction in the CC in both subtypes compared with HC matches existing reports of reduced FA levels in this tract as one of the most robust effects in BD [19, 22, 74]. As a crucial connecting pathway between brain hemispheres, the CC plays a central role in interhemispheric integration. Meta-analyses [75–78] have shown reduced WM integrity in the CC across MDD, BD, and SZ, suggesting disruptions in WM interhemispheric connectivity as a common pathophysiological pathway in major mental disorders, being related to deficits in various emotional and cognitive processes, such as executive functions, attention, working and visual memory [75, 77, 79–81]. Specifically, alterations in the forceps minor of the CC, composed of fibers extending laterally from the genu of the CC and connecting the cerebral hemispheres anteriorly, primarily indicate impaired interhemispheric communication within the prefrontal cortex, whose involvement in BD has been elaborated in several studies e.g. ref. [82–84].

The GMV analyses yielded no significant group effect, refuting our hypothesis. This aligns with findings from two large-cohort studies by the ENIGMA bipolar consortium [20, 21], indicating no subtype-specific (sub-)cortical GMVs differences. In contrast, prior literature found subtype-specific abnormalities [33, 37, 40, 42, 44], but results varied, potentially due to factors such as sample characteristics (e.g. small sample sizes or current mood state) or methodological differences (e.g. voxel-wise comparisons vs. region-specific parcellation). Our study adopted a whole-brain approach to capture this heterogeneity comprehensively, which, given a more stringent statistical threshold, could explain the lack of subtype-specific effects. Accordingly, the uncorrected exploratory analyses (Table S3) suggest the anticipated pattern of BD-I < BD-II < HC in GMVs in parietal, frontal, occipital and parahippocampal/fusiform areas. These regions partly overlap with reported regions in studies reporting subtype-specific differences e.g. [37, 40]. Neurobiological disparities in GMV between BD-I and BD-II may be less distinct than previously thought, necessitating larger, more homogeneous samples for precise investigations and mapping of potential effects. Given this evidence, the brain structural alterations in BD and its subtypes appear to relate less to the volume of structurally independent areas than to impaired fiber connections between these brain regions.

We found significantly lower FA in BD-I compared with BD-II, accompanied by global FA alterations in BD-I compared with HC. The separate diagnosis of BD-II was amended in DSM-IV [85] to acknowledge the disorder’s heterogeneity and to classify less severe symptoms of mania. The significant neurobiological differences between BD-I and BD-II initially support this categorical classification into subtypes [45]. However, the strong distributional overlap in FA reductions between both subtypes challenges a clear-cut classification based on the extent of WM alterations (Fig. 1B). From the latter argument, our data rather suggest a bipolar spectrum with varying severity [46, 47], mirrored by gradual transitions at the clinical level. Only differences in a few criteria and also subjective assessments determine which subtype a patient is diagnosed with [11, 45, 47]. The distinction between bipolar subtypes may be clearer in the presence of critical severity markers, e.g., psychotic symptoms or hospitalization [47], where subtypes differed in our sample (Table 1). However, our dimensional regression analyses revealed no significant linear associations between brain structure and psychotic symptoms, contrasting previous findings [86–88]. Subtype differences were largely unaffected by various variables, although single effects lost significance when controlling for body mass index, PRS or psychotic symptomatology, suggesting a more detailed analysis of these variables in future studies. Nevertheless, these factors provide no obvious explanation for the subtype differences found and challenge the notion of neuroprogressive effects in BD [89–92]. Given this, we can only cautiously interpret our results of distinct subtypes as a neurobiological correlate of overall disease severity in BD-I versus BD-II.

Considering the overlap in genetic compositions and heritability of BD [15], we examined associations between brain structure and PRS for BD. While we observed a significant difference in PRS between subtypes (Table 1), it was not related to gray or white matter features. The PRS employed in this study [15], predominantly derived from BD-I patients, may contribute to the substantial difference observed between subtypes. The debate surrounds the suitability of genome-wide PRS as a genetic tool for investigating psychiatric disorders [93]. While it enhances generalizability by capturing a broad range of common genetic factors for BD [55], its inclusion of alleles with potentially diverse associations with brain structure may mask effects of specific gene sets [93]. This debate is reflected in conflicting findings: On the one hand, there are studies failing to establish significant associations between BD or SZ PRS and brain structure [35, 93]. In contrast, a recent study in bipolar adolescents, benefiting from fewer confounding factors such as illness course or medication, demonstrated higher bipolar PRS scores correlating with both GM structure and WM diffusion [55].

Overall, the inclusion of larger BD-II samples, deep phenotyping, and the investigation of more specific genetic associations may provide relevant insights into the genetic composition of subtypes and putative relationships to brain structural features [94, 95].

This study has several strengths: A large, well-characterized sample of BD patients and comprehensive analyses including clinical, genetic and neuroimaging data. A few limitations are: First, the cross-sectional nature of our analyses entails interpretative difficulties and lower statistical power than longitudinal data. Particularly, the question whether neurobiological abnormalities are a result or precursor for bipolar subtype cannot be addressed. Second, the cross-sectional acquisition of information on previous disease course by self-reports is often biased by inaccuracies and memory deficits [96–98]. Therefore, the regression analyses including these clinical variables should be interpreted cautiously. Third, when using a composite score to represent psychopharmacological treatment, we did consider current medication, but without taking medication history into account, so that possible effects of previous medication use or duration of use cannot be excluded. Fourth, while TBSS offers the advantage of fully automated voxel-wise analysis of the whole brain, revealing changes beyond predefined pathways, the addition of newer tractography techniques may be beneficial for hypothesis-driven identification and detailed analysis of specific pathways [70, 99]. Fifth, we have examined only a selection of dimensions of psychopathology relevant to BD subtypes, but the inclusion of other dimensions, such as affective lability [100, 101], emotion regulation deficits [102], or affective temperaments [103], could also provide important insights. Sixth, we decided to include site as a covariate to control for potential scanner effects between Marburg und Münster as suggested by [63]. However, this approach may over- or under-correct for the site effect and only accounts for linear mean differences [104]. Other approaches of data harmonization between different scanners (e.g. ComBat harmonization) could be discussed as an alternative [105].

In summary, BD-I showed widespread alterations in WM connectivity, with no subtype-specific effects on GMV. The results suggest that BD-I phenomenology may be related to brain structural integrity impairment, which seems mainly independent from disease trajectories and genetics. Our findings may improve our understanding of the pathophysiology underlying the clinical and neurobiological spectrum of BD. Thus, microstructural alterations should be included when discussing the categorical or dimensional classification of bipolar subtypes. Similarly, clinicians may consider the degree of impairment in microstructural integrity when classifying BD patients within the spectrum of affective disorders and adjust treatment selection accordingly, although no potential cutoff regarding the extent of WM alterations has yet been established. Future studies should investigate other biological correlates of BD, for example functional connectivity or inflammatory processes, by more consistently taking subtypes into account.

### Supplementary information

Supplementary Material

Supplementary Table S1

Supplementary Table S2

Supplementary Table S4

Supplementary Table S5

Supplementary Table S6
