# Supplementary material for: White and gray matter alterations in bipolar I and bipolar II disorder subtypes compared with healthy controls – exploring associations with disease course and polygenic risk
Source: Neuropsychopharmacology. 2024 Feb 8;49(5):814–23. doi: 10.1038/s41386-024-01812-7 (PMC10948847; doi:10.1038/s41386-024-01812-7)
Supplement: Supplementary file 1 — Supplementary Material [file 41386_2024_1812_MOESM1_ESM.pdf]

Supplemental Material

Supplement 1: Detailed Information on the Medication Load Index

Current psychopharmacological medication intake was assessed through a previously used composite score, the medication load index.[1] Each psychopharmacological agent was assigned a value between 0 and 2 depending on whether that medication was absent (=0), the dose was equal to or lower than the average dose (low=1) or the dose was higher than average (high=2). The average dose was defined by the average daily dose range recommended by the Physician's-Desk-Reference. By calculating the sum of all medication scores per participant, the resulting score reflected both the number and daily dose of all current psychopharmacological medications taken by the participant.

Supplement 2: Genotyping and Polygenic Risk Scores (PRS)

Biomaterial collection, DNA extraction, genotyping, quality control and imputation have been performed in the larger FOR2107 cohort,[2] of which the current sample is a subset, and have been described in detail elsewhere.[3,4] Briefly, blood samples were acquired at both study sites to extract genomic DNA. According to standard protocols, further preparation and genotyping was performed using the Infinium PsychArray BeadChip (Illumina, San Diego, USA) at the Institute of Human Genetics (University of Bonn, Germany). By using the Genotyping Module (version 1.9.4) implemented in the GenomeStudio software (version 2011.1; Illumina, San Diego, USA), initial quality checks and clustering were performed. Further quality control was conducted using PLINK (version 1.90b5)[5] as published in previous studies.[3,4] Briefly, samples were excluded based on the following criteria: Relatedness between participants with $\hat{\pi} \geq 12.5\%$, genetic duplicates, sex mismatches or other X-chromosome related issues, genotyping call rate <98%, and genetic outlier with a distance from the mean of >4SD in the first eight ancestry

components. Further, variants with a minor allele frequency (MAF) <.1% or Hardy-Weinberg equilibrium (HWE) $p<1\times10\text{-}6$ or variants mapping to the extended MHC region (chromosome 6, 25-35 Mbp) or to a typical inversion site on chromosome 8 /7-13 Mbp) were removed. Multi-dimensional scaling (MDS) components were estimated on genotype data to adjust for population stratification. More specifically, MDS components were estimated based on the identity-by-state distance matrix of all individuals included in this study by using the eigendecomposition-based algorithm in PLINK (version 1.9). According to the scree plot, the first three MDS components were included as covariates in the following analyses.
PRS were calculated with PRS-CS[6] in PLINK based on a previous genome-wide association study (GWAS) for bipolar disorder.[7] Following a study by Kalman and colleagues,[8] we estimated PRS weights with PRS-CS-auto ($\varphi=1.29\times10\text{-}4$). Linear regression analyses were performed between PRS and gray and white matter using SPM and FSL, respectively. As covariates, age, sex, scanner settings (Marburg Body-Coil pre, Marburg Body-Coil post, Münster) and the first three MDS components were included.

Supplement 3: DTI data acquisition, preprocessing and analysis

DTI data acquisition

Fifty-six axial slices with no gap were measured with an isotropic voxel size of $2.5\text{x}2.5\text{x}2.5\text{mm}^3$ (TE=90ms, TS=7300ms), using a GRAPPA acceleration factor of 2. Five non-diffusion-weighted (DW) images (b=0s/mm2) and 2x30 DW images with a b-value of 1000s/mm^2 were acquired. For quality assurance of the data, the open-source software DTIPrep (Oguz et al., 2014) was used with default options. In case of artifacts, individual images of a given participant were eliminated, and a participant was excluded from further analyses if more than 20% of images were affected. On average, the included participants had 64.11 images (SD=1.40, range: 56-65).

DTI data preprocessing

Preprocessing was implemented in FSL6.0.1 (http://fsl.fmrib.ox.ac.uk/fsl/fslwiki/).[9–11] The DW images were corrected for head motion and eddy current induced distortions using "eddy" from FSL,[12] and b-vectors were rotated accordingly. After removal of non-brain tissue using the Brain Extraction Tool (BET) in FSL,[13] the first b0 image from each participant was used as reference for alignment.

DTI data analysis

Analysis of DTI data was performed using TBSS,[14] a technique designed to reduce registration misalignment. Using FMRIB's non-linear registration tool, all FA images were aligned to the FMRIB58 FA template brain in 1×1×1 mm^3 Montreal Neurological Institute (MNI) standard space. All registered FA images were averaged and a threshold of 0.2 was applied to create a WM skeleton representing the centers of the tracts common to all participants. Each participant's aligned FA data were then projected onto the mean skeleton mask by searching for maximum FA values perpendicular to the local skeleton direction.

Calculation of DTI metrics

FA is a measure of the directionality of water diffusion on a scale from 0 (indicating isotropic diffusion) to 1 (indicating completely anisotropic diffusion). It is calculated as the normalized variance of the three eigenvalues. MD represents average water diffusion and is calculated as the mean of the three eigenvalues. AD and RD are specific measures of diffusivity parallel and perpendicular to the principal direction of the axonal fibres, respectively. AD is equivalent to the first eigenvalue, whereas RD is calculated as the average of the second and third eigenvalues.[15,16]

Supplement 4: T1 data acquisition for GMV analyses

Sequence parameters in Münster were 192 sagittal slices with 0.5mm slice gap, TR=2130ms, TE=2.28ms, inversion time=900ms and FA=8° resulting in a voxel size of 1x1x1mm³. In Marburg parameters were 176 sagittal slices with 0.5mm slice gap, TR=1900ms, TE=2.26ms, inversion time=900ms and flip angle=9° resulting in a voxel size of 1x1x1mm³.

Supplement 5: Effects in RD, MD and AD

Analysis 1: HC vs. BD-I vs. BD-II

A significant main effect of diagnosis across HC, BD-I and BD-II was found in RD ($p_{tfce\text{-}FWE}$ =.001, total k=10721 voxels, most affected tracts: forceps minor of the corpus callosum, right anterior thalamic radiation), as well as in MD ($p_{tfce\text{-}FWE}$ =.012, total k=1526 voxels, mainly located in the forceps minor). No significant main effect of diagnosis emerged for AD ($p_{tfce\text{-}FWE}$=.398). Pairwise post-hoc *t*-tests revealed significantly increased RD values in BD-I patients compared with BD-II (*d*=0.41, $p_{tfce\text{-}FWE}$ =.028, total k=1970 voxels, mainly localized in the forceps minor) as well as HC (*d*=0.27, $p_{tfce\text{-}FWE}$ =.001, total k=36957 voxels, most affected tracts: forceps minor and right superior longitudinal fasciculus), but no difference between BD-II and HC ($p_{tfce\text{-}FWE}$ =.074). For MD, only the difference between BD-I and HC reached significance (*d*=0.28, $p_{tfce\text{-}FWE}$ =.001, total k=36957 voxels), but there were no significant differences between BD-II and HC ($p_{tfce\text{-}FWE}$ =.125) or BD-I and BD-II ($p_{tfce\text{-}FWE}$ =.067) (see Tables S1 and S2).

Analysis 2: Additional analyses

a) Brain Structural differences between BD-I and BD-II subtypes correcting for clinical variables and polygenic risk

Due to non-significant differences between BD-I and BD-II patients in AD and MD in analysis 1, this analysis was calculated for RD only. Analyses revealed a stable increase of RD in BD-I vs. BD-II even when additionally correcting for number of depressive episodes (*d*=0.47, $p_{\text{tfce-}}$

$_{FWE}$=.035, *k*=1215 voxels), number of (hypo-)manic episodes (*d*=0.54, $p_{tfce\text{-}FWE}$=.042, *k*=379 voxels), number of psychiatric hospitalizations (*d*=0.37, $p_{tfce\text{-}FWE}$=.027, *k*=3903 voxels), time since first symptoms (*d*=0.42, $p_{tfce\text{-}FWE}$=.03, *k*=1782 voxels), time since first psychiatric hospitalization (*d*=0.36, $p_{tfce\text{-}FWE}$=.023, *k*=5957 voxels), and childhood adversity (d=0.43, $p_{tfce\text{-}FWE}$=.026, *k*=1872 voxels). Similarly, the observed pattern of results did not change when correcting the model for medication load (*d*=0.40, $p_{tfce\text{-}FWE}$=.026, *k*=2515 voxels) (see Table S1). However, with the additional correction for PRS for BD, the difference between subtypes was no longer found to be significant ($p_{tfce\text{-}FWE}$=.072), which was also the case with the additional correction for BMI ($p_{tfce\text{-}FWE}$=.107) and lifetime psychotic symptoms ($p_{tfce\text{-}FWE}$=.056). The most affected tract was consistently the forceps minor (Table S2).

b) Associations with clinical variables and polygenic risk within all BD patients

For RD, MD, and AD, BD patients showed a negative association with the time since first psychiatric hospitalization (RD: $p_{tfce\text{-}FWE}$ =.006, k=45300 voxels; MD: $p_{tfce\text{-}FWE}$ =.003, k=56027; AD: $p_{tfce\text{-}FWE}$ =.009, k=10951). Furthermore, AD showed a negative association with body mass index ($p_{tfce\text{-}FWE}$ =.032, k=440) (Table S1 and S2). However, only the association between MD and time since first psychiatric hospitalization survived Bonferroni correction. Beyond that, no significant associations emerged between clinical and genetic features and AD, MD, or RD.

Supplementary Tables

Table S1: Cluster sizes and MNI coordinates of the peak voxel of all significant clusters, derived with the "cluster" tool implemented in FSL. (see separate excel file)

Table S2: Anatomical regions comprising the significant effects of the analyses based on the "JHU White-Matter Tractography Atlas". (see separate excel file)

Table S3.

Results of the exploratory whole-brain analyses of gray matter volumes, pairwise comparisons between HC, BD-I and BD-II, conducted at p<.001, uncorrected, with a threshold of k=50.

Anatomic Label	Hemisphere	k	x	y	z	*t*-Score
Contrast: HC>BD-I						
Inferior and superior parietal gyrus	R	184	45	-42	52	*4.29*
Middle cingulate gyrus, superior motor area	R	464	12	-3	52	*4.18*
Lingual gyrus, cerebellum crus 6	L	626	-18	-36	-10	*4*
Middle frontal gyrus	R	133	32	33	39	*3.75*
Fusiform gyrus	R	59	20	0	-4	*3.55*
Parahippocampal gyrus, fusiform gyrus	R	81	36	-21	-41	*3.41*
Contrast: HC>BD-II						
Middle frontal gyrus	L	57	-36	57	14	*3.38*
Contrast: BD-II>BD-I						
Superior motor area	R	118	4	-3	52	*3.65*
Middle occipital gyrus	R	63	27	-80	14	*3.52*
Rolandic operculum, insula	L	59	-33	-27	-16	*3.23*
Contrast: BD-I>BD-II						
-	-	-	-	-	-	-

Abbreviations: L=Left, R=Right, BD-I=bipolar 1 subtype, BD-II=bipolar 2 subtype, HC=healthy controls.

Table S4: Difference between BD-I and BD-II in fractional anisotropy and radial diffusivity, corrected for different types of medication. (see separate excel file)

Table S5: Associations between clinical variables and diffusion tensor imaging metrics in BD-I and BD-II separately (see separate excel file)

Table S6: Associations between clinical variables and GMV in BD-I and BD-II separately (see separate excel file)

References

1. Hassel S, Almeida JRC, Kerr N, et al. Elevated striatal and decreased dorsolateral prefrontal cortical activity in response to emotional stimuli in euthymic bipolar disorder: no associations with psychotropic medication load. Bipolar Disord. 2008;10(8):916-927. doi:10.1111/j.1399-5618.2008.00641.x
2. Kircher T, Wöhr M, Nenadić I, et al. Neurobiology of the major psychoses: a translational perspective on brain structure and function—the FOR2107 consortium. Eur Arch Psychiatry Clin Neurosci. 2019;269(8):949-962. doi:10.1007/s00406-018-0943-x
3. Schmitt S, Meller T, Stein F, et al. Effects of polygenic risk for major mental disorders and cross-disorder on cortical complexity. Psychol Med. 2021;(Mdd):1-12. doi:10.1017/S0033291721001082
4. Meller T, Schmitt S, Stein F, et al. Associations of schizophrenia risk genes ZNF804A and CACNA1C with schizotypy and modulation of attention in healthy subjects. Schizophr Res. 2019;208(xxxx):67-75. doi:10.1016/j.schres.2019.04.018
5. Chang CC, Chow CC, Tellier LCAM, Vattikuti S, Purcell SM, Lee JJ. Second-generation PLINK: rising to the challenge of larger and richer datasets. Gigascience. 2015;4(1):7. doi:10.1186/s13742-015-0047-8
6. Ge T, Chen C-Y, Ni Y, Feng Y-CA, Smoller JW. Polygenic prediction via Bayesian regression and continuous shrinkage priors. Nat Commun. 2019;10(1):1776. doi:10.1038/s41467-019-09718-5
7. Mullins N, Forstner AJ, O'Connell KS, et al. Genome-wide association study of more than 40,000 bipolar disorder cases provides new insights into the underlying biology. Nat Genet. 2021;53(6):817-829. doi:10.1038/s41588-021-00857-4
8. Kalman JL, Papiol S, Grigoroiu-Serbanescu M, et al. Genetic risk for psychiatric illness is associated with the number of hospitalizations of bipolar disorder patients. J Affect Disord. 2022;296:532-540. doi:10.1016/j.jad.2021.09.073
9. Jenkinson M, Beckmann CF, Behrens TEJ, Woolrich MW, Smith SM. FSL. Neuroimage. 2012;62(2):782-790. doi:10.1016/j.neuroimage.2011.09.015
10. Smith SM, Jenkinson M, Woolrich MW, et al. Advances in functional and structural MR image analysis and implementation as FSL. Neuroimage. 2004;23(Suppl 1):S208-19. doi:10.1016/j.neuroimage.2004.07.051
11. Woolrich MW, Jbabdi S, Patenaude B, et al. Bayesian analysis of neuroimaging data in FSL. Neuroimage. 2009;45(1 Suppl):S173-86. doi:10.1016/j.neuroimage.2008.10.055
12. Andersson JLR, Sotiropoulos SN. An integrated approach to correction for off-resonance effects and subject movement in diffusion MR imaging. Neuroimage. 2016;125:1063-1078. doi:10.1016/j.neuroimage.2015.10.019
13. Smith SM. Fast robust automated brain extraction. Hum Brain Mapp. 2002;17(3):143-155. doi:10.1002/hbm.10062
14. Smith SM, Jenkinson M, Johansen-Berg H, et al. Tract-based spatial statistics: Voxelwise analysis of multi-subject diffusion data. Neuroimage. 2006;31(4):1487-1505. doi:10.1016/j.neuroimage.2006.02.024

15. Feldman HM, Yeatman JD, Lee ES, Barde LHF, Gaman-Bean S. Diffusion tensor imaging: A review for pediatric researchers and clinicians. J Dev Behav Pediatr. 2010;31(4):346-356. doi:10.1097/DBP.0b013e3181dcaa8b

16. Alexander AL, Lee JE, Lazar M, Field AS. Diffusion Tensor Imaging of the Brain. Neurotherapeutics. 2007;4(3):316-329. doi:10.1016/j.nurt.2007.05.011

17. Wakana S, Caprihan A, Panzenboeck MM, Fallon JH, Perry M, Gollub RL, et al. (2007): Reproducibility of quantitative tractography methods applied to cerebral white matter. Neuroimage 36: 630–644.

18. Mori S, Wakana S, van Zijl P, Nagae-Poetscher L (2005): MRI Atlas of Human White Matter, 1st ed. Amsterdam, The Netherlands: Elsevier.

19. Hua K, Zhang J, Wakana S, Jiang H, Li X, Reich DS, et al. (2008): Tract probability maps in stereotaxic spaces: Analyses of white matter anatomy and tract-specific quantification. Neuroimage 39: 336–347.
